# Complications after surgery for malignant melanoma do not delay further treatment

**DOI:** 10.1007/s00238-021-01839-9

**Published:** 2021-10-05

**Authors:** Sara Munkhammar, Carl Sars, Inkeri Schultz, Peter Gillgren, Ebba K. Lindqvist

**Affiliations:** 1 Division of Reconstructive Plastic Surgery, Department of Molecular Medicine and Surgery, Karolinska Institutet, 171 77 Stockholm, Sweden; 2 Reconstructive Plastic Surgery, Karolinska University Hospital, 171 76 Stockholm, Sweden; 3 Department of Surgery, South General Hospital, 118 83 Stockholm, Sweden; 4 Department of Clinical Science and Education, Karolinska Institutet, 171 77 Stockholm, Sweden

Sentinel lymph node biopsy (SNB) is commonly employed in malignant melanoma and has previously routinely been followed by a completion lymph node dissection (CLND) with the purposes of achieving local cancer control and minimizing the risk of further spread. Surgical complications after SNB occur in approximately 12% of patients and include seroma, wound infection, lymphoedema, hematoma, and nerve injury [1]. These complications may also occur after CLND, and the overall complication rate is significantly higher after this procedure due to its extensive nature [2].

Post-operative complications have the potential to cause an undesirable delay in subsequent treatment, since complications such as wound dehiscence and infection can be contraindications to adjuvant therapy. Recent studies have showed that post-operative complications after surgical procedures may not only delay further oncological treatment but even prohibit the otherwise planned treatment [3–5]. In these studies, the researchers concluded that timely initiation of appropriate treatment is favorable for overall survival [4, 5]. This has not previously been investigated in melanoma.

We conducted a retrospective cohort study to determine whether complications due to SNB cause a delay to CLND and whether complications due to CLND cause a delay in adjuvant oncological treatment, for patients with malignant melanoma. We included all patients with melanoma who underwent CLND in the Stockholm region from 2006 to 2018. Details on patient characteristics, tumor type, treatment, and post-operative complications were collected from medical records. The mean times between SNB and CLND, and between CLND and adjuvant treatment, were compared between patients with and without post-operative complications. Time to treatment was measured in days, and mean time to treatment was compared between the two treatment groups using a two-tailed *t* test. Statistical analysis was carried out using Stata version 13.1. Results were presented as means with standard deviations and as odds ratios (OR) with a 95% confidence interval (CI). A *p* value ≤ 0.05 was considered statistically significant. The Regional Ethical Review Board in Stockholm approved this study (reference numbers 2017/795–31 and 2018/1853–32).

We identified 232 patients who underwent a CLND during the study period. Firstly, we looked at time to CLND in patients with a positive SNB for malignant melanoma, compared between patients with and without post-operative complications after SNB. Patients who underwent therapeutic lymph node dissection (TLND) for malignant melanoma not due to a positive SNB but due to a clinically or radiologically detected metastasis were excluded from this analysis which included 180 patients in total (Table 1). The mean time between SNB and subsequent CLND was 44 days. Due to unspecified complications and/or inaccessible data, an additional 17 patients were excluded from further analysis. Details on post-operative complications after sentinel lymph node biopsy in our cohort have been published previously [1]. The most common complications were infection and seroma, followed by wound dehiscence, hematoma, lymphoedema, and other complication [1]. The mean period between SNB and CLND was 45 days for patients with post-operative complications after SNB and 44 days for patients without post-operative complications (*p* = 0.766).

**Table 1:** **Treatment information**. One hundred eighty patients who underwent sentinel node biopsy (SNB) and subsequent completion lymph node dissection (CLND)

Variable	Number (frequency or range)
Sex	
Male	112 (62.2%)
Female	68 (37.8%)
Mean age, years (range)	55.4 (23–75)
Site of SNB	
Axilla	100 (55.6%)
Inguinal	80 (44.4%)
SNB complications	
Yes	26 (14.4%)
No	137 (76.1%)
Unknown	17 (9.4%)
SNB to CLND in days, mean (range)	44.4 (0–131)

Abbreviations: *SNB* sentinel node biopsy, *CLND* completion lymph node dissection

Secondly, we examined time to further oncological treatment in patients after CLND for malignant melanoma, compared between patients with and without post-operative complications after CLND. Patients who did not have adjuvant treatment in the form of immunotherapy, chemotherapy, or radiotherapy within 6 months of CLND were excluded from this analysis which included 31 patients in total (Table 2). The most common complications after CLND in our cohort were lymphoedema, infection, and seroma, followed by wound dehiscence and other complications, as detailed previously [2]. The mean time to treatment between CLND and adjuvant treatment was 97 days. All patients had accessible data on complications and adjuvant treatment. The mean period between CLND and adjuvant treatment was 95 days for patients with post-operative complications after CLND and 100 days for patients without post-operative complications (*p* = 0.739). We were not able to identify any patient who was planned for adjuvant therapy within 6 months but had treatment delay past 6 months due to complications after CLND.

**Table 2:** **Treatment information.** Thirty-one patients who underwent completion lymph node dissection (CLND) and received adjuvant treatment within six months

Variable	Number (frequency or range)
Sex	
Male	23 (74.2%)
Female	8 (25.8%)
Mean Age, years (range)	56.2 (26–82)
Positive CLND	
Yes	14 (45.2%)
No	6 (19.3%)
TLND was done due to metastasis*	11 (35.5%)
Complications	
Yes	15 (48.4%)
No	16 (51.6%)
Adjuvant treatment	
Radiotherapy	7 (22.6%)
T-cell activated antibodies	10 (32.2%)
Kinase inhibitors; BRAF-inhib., MEK-inhib	6 (19.4%)
Other chemotherapy (e.g. Temozolomide)	6 (19.4%)
Regional perfusion therapy	1 (3.2%)
Radio therapy + chemotherapy	1 (3.2%)
CLND to Onc treatm. in days, mean (range)	96.9 (23–213)

^*^Detected through clinical examination or radiology

Abbreviations: *CLND* Completion lymph node dissection, *TLND* Therapeutic lymph node dissection, *BRAF* v-Raf murine sarcoma viral oncogene homolog B1, *MEK* Mitogen-activated protein kinase kinase

In conclusion, in this retrospective cohort study, we show that surgical complications do not appear to cause a delay in melanoma treatment, either from SNB to CLND or from CLND to adjuvant treatment when indicated. Studies in breast cancer and colorectal cancer have demonstrated delays of adjuvant therapy or worse overall survival, in patients with post-operative complications [3–5]. Considering the metastatic potential of melanoma disease, it can be assumed that timely treatment is associated with better outcomes. However, contrary to for example colorectal cancer surgery, post-operative complications due to melanoma surgery are largely at the surgical site and not systemic. It is possible that post-operative complications due to SNB or CLND for melanoma either resolve quickly enough not to delay further treatment or are not perceived by treating physicians as contraindications for further treatment.

To the best of our knowledge, this is the first study on this patient group describing time to treatment and examining the potential influence of post-operative complications in patients with malignant melanoma. Although perhaps somewhat limited by small numbers and by the regional and retrospective study design, we believe the conclusion of our study must be that post-operative complications after surgery for malignant melanoma do not need to be a great concern when it comes to subsequent treatment.
